# Enhancement of Biomass Production of Diatom *Nitzschia* sp. S5 through Optimisation of Growth Medium Composition and Fed-Batch Cultivation

**DOI:** 10.3390/md22010046

**Published:** 2024-01-17

**Authors:** Marina Grubišić, Božidar Šantek, Marija Kuzmić, Rozelindra Čož-Rakovac, Mirela Ivančić Šantek

**Affiliations:** 1 Faculty of Food Technology and Biotechnology, University of Zagreb, 10000 Zagreb, Croatia; marina.grubsic22@gmail.com (M.G.); bsantek@pbf.hr (B.Š.); marija.kuzmic56@gmail.com (M.K.); 2 Laboratory for Aquaculture Biotechnology, Division of Materials Chemistry, Ruđer Bošković Institute, 10000 Zagreb, Croatia; rozelindra.coz-rakovac@irb.hr; 3 Center of Excellence for Marine Bioprospecting (BioProCro), Ruđer Bošković Institute, 10000 Zagreb, Croatia

**Keywords:** marine microalgae, diatoms, biomass production, nitrogen, silicon, phosphate, nutrient concentration ratio, fed-batch cultivation

## Abstract

The growing commercial application of microalgae in different industry sectors, including the production of bioenergy, pharmaceuticals, nutraceuticals, chemicals, feed, and food, demands large quantities of microalgal biomass with specific compositions produced at reasonable prices. Extensive studies have been carried out on the design of new and improvement of current cultivation systems and the optimisation of growth medium composition for high productivity of microalgal biomass. In this study, the concentrations of the main macronutrients, silicon, nitrogen and phosphorus, essential for the growth of diatom *Nitzschia* sp. S5 were optimised to obtain a high biomass concentration. The effect of main macronutrients on growth kinetics and cell composition was also studied. Silicon had the most significant effect on diatom growth during batch cultivation. The concentration of biomass increased 5.45-fold (0.49 g L^−1^) at 1 mM silicon concentration in modified growth medium compared to the original Guillard f/2 medium. Optimisation of silicon, nitrogen, and phosphorus quantities and ratios further increased biomass concentration. The molar ratio of Si:N:P = 7:23:1 mol:mol:mol yielded the highest biomass concentration of 0.73 g L^−1^. Finally, the fed-batch diatom cultivation of diatom using an optimised Guillard f/2 growth medium with four additions of concentrated macronutrient solution resulted in 1.63 g L^−1^ of microalgal biomass. The proteins were the most abundant macromolecules in microalgal biomass, with a lower content of carbohydrates and lipids under all studied conditions.

## 1. Introduction

Diatoms are a specific group of photosynthetic microalgae characterised by a siliceous skeleton called a frustule. They are considered cosmopolitan phytoplanktons since they are found in almost every aquatic environment, including fresh and marine waters, with around 200,000 known species [1]. Diatoms are dynamic microorganisms with rich biodiversity due to their ability to adapt to diverse environments, including extreme and oscillating conditions (e.g., solar, osmotic, oxidative and nutrient stress). Microalgae are an outstanding source of diverse substances with potential applications in various industries, including bioenergy, pharmaceutical, nutraceutical, chemical, feed and food [2,3]. Microalgal biomass contains valuable proteins, carbohydrates, and lipids that could be used as food and feed. They also synthesise diverse metabolites with biological activity, including pigments and compounds with anticancer, antibacterial, antiviral, and antiparasitic activity [4]. Moreover, the production of biofuels from sustainable renewable microalgal biomass reduces the emission of CO_2_ [2,3,5,6]. Still, large-scale cultivation of microalgae is constrained by several issues from upstream to downstream processes. One of the main issues connected to cultivation conditions is light availability and insufficient or extensive mixing, resulting in heterogeneity of culture broth and cell death. Open cultivation systems are exposed to increased contamination risk by competing microorganisms and an environmental risk due to the potential threat of releasing nutrients in surroundings, causing eutrophication and releasing the producing strain in the environment, affecting biodiversity. Open cultivation systems also lack online monitoring and control of process parameters, while closed systems are associated with high capital costs, high biomass productivity, and low risk of contamination. Commercial large-scale cultivation microalgae is still economically unfeasible for low-value products such as biofuels due to high investment costs, low biomass concentration and productivity, and high costs of the downstream process (harvesting and dewatering the biomass) [3,6,7,8,9]. Optimisation of cultivation conditions and growth media is mandatory to provide sustainable biomass production in a cost-effective way [10]. Growth deprivation on limiting macronutrients such as silicate, nitrate, and/or phosphate increases the content of triacylglycerides (TAG) in diatoms. However, accumulation of TGA is accompanied by concomitant reduction in biomass productivity due to decreased growth rates under nutrient depravation [7,10]. Moreover, the reduction in production cost is linked not only to lipid productivity but also to biomass productivity [5].

Different approaches have been applied in order to improve the growth of diatoms, including feeding the culture with additional carbon sources by supplementation of air with carbon dioxide (CO_2_) at range from 1 to 15% (vol vol^−1^) [2], addition of sodium bicarbonate [10], as well as different cultivation modes. Generally, various growth media developed for microalgal cultivation contain the same basic components, including macronutrients, nitrogen, phosphorus and silicon, and micronutrients such as trace elements and vitamins (e.g., cobalamin, biotin, and thiamine) [11,12]. One commonly used growth medium for cultivating marine microalgae, especially diatoms, is the Guillard f/2 medium. Although this growth medium provides all three macronutrients needed for diatom growth, the amounts are sufficient for culture maintenance and not for microalgal biomass production. Therefore, the first step in intensifying biomass production is the optimisation of nutrient concentrations in the growth medium. The present work aimed to enhance the growth of diatom *Nitzschia* sp. S5 through optimisation of Guillard f/2 growth medium. In a previous study, the fast-growing diatom *Nitzschia* sp. S5 was isolated from the Adriatic Sea, purified, and characterised for potential biotechnological application. Under unoptimised conditions, *Nitzschia* sp. S5 accumulates lipids with a high content of neutral lipids, which are generally regarded as the most valuable lipid fraction for biofuel production. Along with high-value long-chain polyunsaturated fatty acids, this diatom strain produces significant amounts of pigments, of which fucoxanthin and lutein are the most abundant. Furthermore, cell extract shows strong antioxidant and antimicrobial activity against several bacterial and yeast strains. Due to high protein content, *Nitzschia* sp. S5 could be an interesting feedstock for food and feed production [13]. The first objective of this work was to evaluate the effect of nutrient concentration on the growth and biomass composition of diatom *Nitzschia* sp. S5. The molar ratio of these macronutrients is also crucial for diatom growth and differs from one strain to another. Hence, the second objective was to determine the optimal molar ratio of studied macronutrients for obtaining the high biomass production. The optimised growth medium was used in the fed-batch cultivation of *Nitzschia* sp. S5.

## 2. Results and Discussion

### 2.1. Effect of Nitrogen, Phosphorus, and Silicon Concentration on Growth and Biomass Composition

Maintaining optimal growth conditions and nutrient supply is critical for obtaining high biomass productivity in large-scale cultivations. Nitrogen, phosphorus, and silicon are the major constituents of diatom biomass and, therefore, the most abundant macronutrients in the f/2 growth medium. However, these nutrients are often first exhausted during cultivation. Firstly, the effect of these macronutrients on the growth of *Nitzschia* sp. S5 was investigated. Diatom growth was assessed by the number of cells grown per mL of cell culture (Supplementary Materials, Figures S1–S3).

Since phytoplankton species follow specific diel periodicity in cell division and nitrogen, phosphorus, and silicon uptake rates, the culture was sampled 2–3 h after the photoperiod [14,15,16,17]. The concentration range of each macronutrient was chosen based on literature data and solubility in f/2 medium [11,12,18,19,20]. Due to the low solubility of metasilicate in seawater (1.5 to 1.7 mM at 25 °C [21]), the maximal silicon concentration in the experiment was 2 mM. Although the phosphorus content in microalgal biomass is less than 1%, this element is one of the limiting substrates in microalgal cultivation. The main reason is that phosphorus precipitates by complexation with metal ions present in growth media such as calcium, magnesium, and iron, especially under slightly alkaline conditions [11,12].

Characteristic growth curves for the growth of single-cell microorganisms with the lag, exponential, and stationary phases were obtained. The duration of certain phases depended on the concentration of the studied macronutrients. Table 1 presents growth characteristics as well as lipid and biomass productivities for the growth of *Nitzschia* sp. S5.

The specific growth rate is controlled by nutrient concentration, light intensity, temperature, pH, salinity, oxygen, and surrounding cells (i.e., self-shading) [22,23,24]. For all three limiting macronutrients, values of growth rates of *Nitzschia* sp. S5 followed a similar trend. A positive correlation between concentration and the growth rate was observed at a lower concentration range, while at higher macronutrient concentrations, the growth rate declined. Furthermore, in the absence of nitrogen, silicon, and phosphorus sources, the growth rates were relatively high compared to maximal values obtained for all three macronutrients (Table 1). Growth in the absence of phosphate and nitrogen can be explained by cell reserve of these macronutrients in the microalgal cell [25,26,27]. Diatoms have a large vacuole for hoarding the nutrient excess under nutrient-replete growth conditions. Phosphorus and nitrogen reserves enable microalgae to divide for several generations without an external source until these reserves run out after longer-term starvation. Therefore, Droop et al. suggested relating the growth rate to the intracellular concentration of nitrogen and phosphorus instead of the external concentration [28,29]. Weak growth was also observed in the growth medium without added silicon. The negligible growth under these conditions could be supported by intracellular silicon storage pools and silicon from artificial seawater used for growth medium preparation [30]. Glass Erlenmeyer flasks usually used for microorganism cultivation provide limited quantity of this macronutrient through the effect of silicon leaching from borosilicate glass. In order to examine the contribution of leached silicon to total cell growth, cultivations without added silicon were also performed in plastic Erlenmeyer flasks. The dry cell concentration was roughly cut in half and the growth rate decreased fivefold, suggesting that the leaching of silicon from the glass Erlenmeyer flask provides a noticeable quantity of silicon for diatom growth.

A decrease in growth rate at higher concentrations was observed for all three macronutrients, suggesting growth inhibition kinetics (Table 1). Most studies on nitrogen and phosphorus uptake rates in various diatom species were conducted under relatively low concentrations, and growth followed Monod kinetics [20]. Since the inadequate nutrient supply reduces the performance of microalgae cells, the concentration of macronutrients is usually in excess, i.e., at saturation concentration to provide maximal productivity and growth. Typical substrate inhibition kinetics were observed for several microalgae at high nitrogen and phosphate concentrations in several studies. Metsoviti et al. observed a maximal growth rate in green microalgae *Chlorella vulgaris* at 200 mg L^−1^ (14.3 mM) of nitrogen and a gradual decrease in growth above 400 mg L^−1^ (28.6 mM) [31]. Diatoms are more susceptible to nitrogen inhibition compared to green algae. The maximal specific growth rate of *Nitzschia* sp. S5 in this study was obtained at significantly lower nitrogen concentrations of 3.5 mM (49 mg L^−1^) followed by a similar trend of gradual decrease with further increase in concentration. Obtained values were comparable to values reported in the literature for diatoms *Phaeodactylum tricornutum* and *Nitzschia longissima* (8.82 mM (123 mg L^−1^) and 3.5 mM (49 mg L^−1^), respectively) [20].

Phosphorus is a minor component of the microalgal cell, and the assimilation efficiency is much lower than for the other studied macronutrients [12]. Since this element is one of the limiting factors in the natural environment, microalgae assimilate more of this element than is required for growth and store it in polyphosphate granules under phosphorus-excess conditions. Phosphorus reserve enables microalgae to divide for several generations without an external source until polyphosphate runs out after longer-term starvation [26,27]. The effect of phosphorus concentration was studied among different microalgal strains and cultivation conditions [32,33]. The inhibitory effect of phosphorus on cell biomass depends on diatom strain, cultivation conditions, and type of carbon/energy source. High phosphorus levels (≥150 mg L^−1^) inhibited heterotrophic growth of *Chlorella regularis* and severely damaged the cell (≥150 mg L^−1^) [33]. According to Martinez et al., the inhibitory phosphorus concentration of phototrophically grown *Scenedesmus obliquus* depends highly on growth temperature, and the inhibitory effect decreases with an increase in temperature [32]. Unlike green microalgae, the studied diatom was more susceptible to phosphate inhibition. Maximal growth rate and biomass concentration for diatom *Nitzschia* sp. S5 was obtained at 0.036 mM phosphorus concentration. However, excessively high phosphate concentrations (>0.036 mM) had a counterproductive effect on cell growth rate. Excess of phosphorus negligibly affected biomass concentrations, although the growth rate was almost reduced by half. Similarly, an increase in phosphorus concentration in a range from 7 to 144 μM also had a positive effect on the specific growth rate and biomass concentration of diatom *Chaetoceros muelleri*, but further increase in phosphorus concentration inhibited cell growth [34].

Among all studied macronutrients, silicon had the most pronounced effect on diatom growth and lipid productivity (Table 1). The highest growth rate of 0.35 day^−1^ was obtained at 1 mM silicon concentration, resulting in the highest final biomass concentration (0.49 g L^−1^) and biomass productivity (37.61 mg L^−1^ day^−1^). Specific growth rates of *Nitzschia* sp. S5 were comparable to benthic diatom *Nitzschia longissima* cultivated at similar silicon concentrations [20]. At a lower silicon concentration range (<1 mM), the growth rates were almost constant, indicating no effect of silicon on cell growth, possibly due to silicon leaching. Contrary to expectation, maximal lipid productivity was obtained under silicon-replete conditions (0.5 mM).

Furthermore, the original composition of the growth medium changed due to exposure to high temperature during autoclaving. Silicon tends to polymerise, so the concentration of the assimilable silicon decreases. If the rate of consumption of the assimilable monomeric and dimeric form of silicon by diatom is higher than their generation by depolymerisation from silicon polymers [35,36], it could limit diatom growth, resulting in a decrease in specific growth rates, the concentration of biomass, and cell number at higher Si concentrations (1.5 and 2 mM). Some authors also observed an inhibitory effect in the presence of high silicon concentration [35].

**Figure 1:**
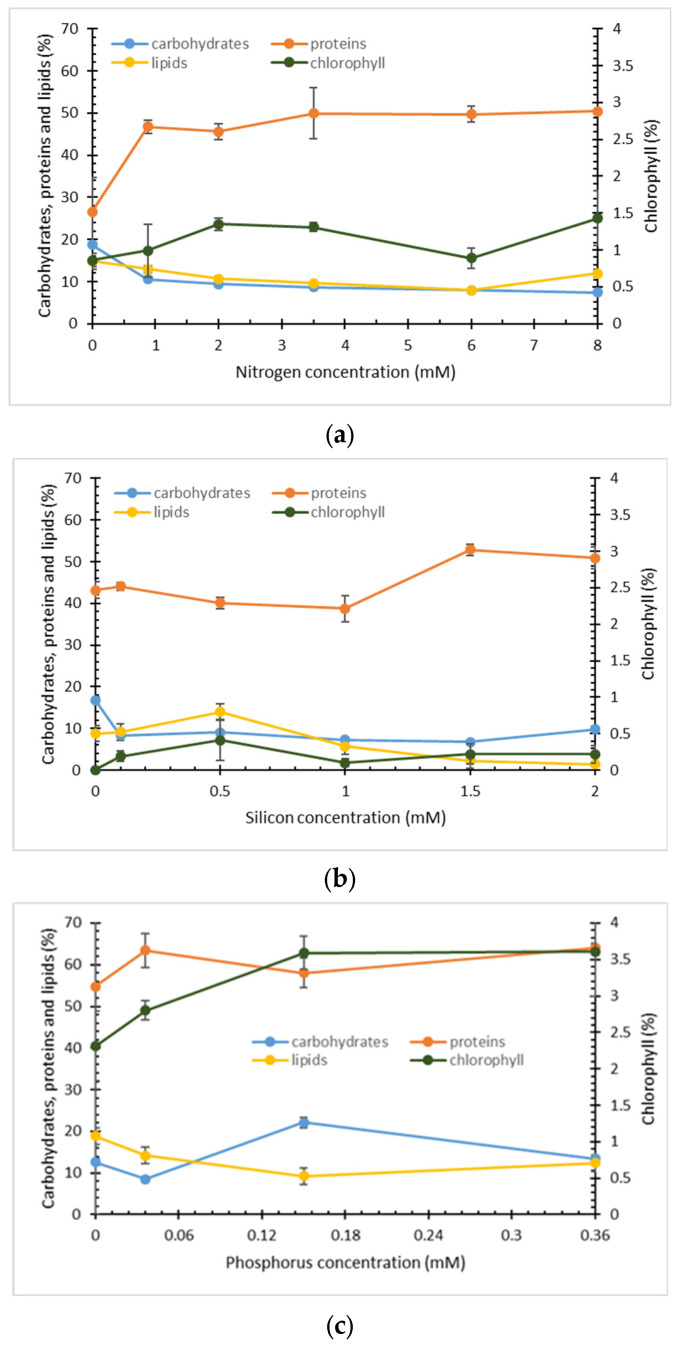
The effect of nitrogen (**a**), silicon (**b**), and phosphorus (**c**) concentration on biomass composition.

Low concentrations of all three nutrients at the end of cultivation under all studied conditions (Table S1; Supplementary Materials) suggest that diatom growth could be simultaneously limited by more than one macronutrient. Thus, the effect of the initial concentration of limiting substrates on growth observed for all three main nutrients could be caused not only by inhibition at high substrate concentration but also by deprivation of growth by another macronutrient. Change in growth medium pH during cultivation, especially at a later growth phase, could also induce precipitation of inorganic macro- and micronutrients. Carbon dioxide also helps to maintain pH at an optimum level. In the later phase of cultivation, the concentration of cell density and the net carbon consumption rate increase, and the concentration of dissolved carbon dioxide in the growth medium reduces, causing pH to rise and encourage precipitation of nutrients [37]. Lipid, protein, and carbohydrate content in microalgal biomass depends on the composition of major macronutrients in the growth medium. A nutrient limitation is a common strategy to induce energy reserve accumulation in various microorganisms. Diatoms store carbon in the form of chrysolaminarin (1,3-ß-glucan) and/or neutral lipids [2,5,7,38]. In this study, the absence of nitrogen did not induce the accumulation of both energy reserves in *Nitzschia* sp. S5 (Figure 1). The content of carbohydrates was higher than lipids (18.69% versus 14.82%) but lower than protein content. Nitrogen is an essential constituent of vital molecules such as amino acids, nucleic acids, and chlorophyll and is an indispensable precursor for protein synthesis [39,40]. Due to nitrogen starvation, protein synthesis declines, diverting carbon lux from protein synthesis to lipid and/or carbohydrate synthesis. Nitrogen deprivation is commonly used to induce lipid accumulation in oleaginous microorganisms. Besides the change in the macromolecular composition of the cell, the lack of nitrogen also decreases the photosynthetic activity in microalgae [41]. The strongest effect of nitrogen starvation is observed on protein content (Figure 1). Under these conditions, the lowest protein content of 26.60% was observed, accompanied by a low growth rate (µ = 0.22 day^−1^, Table 1) in accordance with published studies. An increase in nitrogen concentration in a range from above 0.88 did not significantly affect the protein content in cells (45.59–49.93%). Similar protein content was also observed in other microalgae such as *Isochrysis galbana*, *Scenedesmus* sp., *Chlorella vulgaris*, *Chlorella minutissima*, and *Tetraselmis suecica* [38,42,43,44].

The highest lipid content of 18.83% in this study was obtained in the absence of phosphorus (0 g L^−1^). It is known that microalgae adapt to phosphorus scarcity in several ways. Phosphorus deprivation in microalgae *Phaeodactylum tricornutum* leads to a change in carbon flux in cell metabolism through the induction of the pentose phosphate pathway and gluconeogenesis and repression of the Calvin cycle, resulting in a higher level of lipids, primarily triacylglycerols [39]. In order to maintain growth, phytoplanktons reduce phosphorus requirements by replacing phospholipids in their membrane with non-phosphorus lipids such as sulfolipids in *Thalassiosira pseudonana* [45]. Lipid content in *Nitzschia* sp. S5 was comparable with values reported for *Navicula incerta* and *Nitzschia inconspicua* [46,47].

Similar results were reported by other authors suggesting that under phosphorus starvation conditions, synthesis of proteins would continue if a nitrogen source was available, at least during the early exponential phase [46,48,49,50]. Higher carbohydrate and lipid levels are reported for cultivations supplemented with carbon source, especially CO_2_. Thus, in microalga *Isochrysis galbana* grown under nitrogen-limited conditions supplemented with enriched air with CO_2_ (0.04%, vol vol^−1^), carbohydrate content reached 47.0% [51]. Likewise, aeration of the culture *Nitzschia inconspicua* with CO_2_-enriched air (5% vol vol^−1^) doubled the carbohydrate content and increased lipid content by 50%. Furthermore, the increase in carbohydrate content in the presence of nitrogen and additional carbon (5% CO_2_) source was higher than under nitrogen-limiting conditions. Lipid accumulation was also induced but to a smaller extent than carbohydrates, while nitrogen starvation itself did not affect lipid accumulation [47].

Contrary to several published studies, silicon deficiency in this work did not stimulate the synthesis of lipids in *Nitzschia* sp. S5 [7,36,52]. Furthermore, the excess of silicon (2 mM) resulted in the lowest lipid content of only 1.41%. Several studies investigating the effect of silicon concentration on lipid content showed that diatoms under silicon-limited conditions directly assimilated carbon towards lipid synthesis and, to a smaller extent, to carbohydrate synthesis, producing small amounts of neutral lipids, mainly triacylglycerides (TAG) [53,54]. Growth limitation with carbon source could also be a reason for low lipid synthesis since the cultures were aerated without additional carbon supply. The diatoms grew for several generations due to the diatom’s ability to store cytoplasmic silicon and phosphate reserves and use them under unfavourable conditions [30,35,55]. Comparing the composition of *Nitzschia* sp. S5 with literature data for other diatoms, it can be concluded that the composition of macromolecules depends not only on growth media composition but also on the species of diatoms. The effect of phosphorus limitation on lipid accumulation in several diatom strains was described in the literature. Phosphorus limitation stimulated lipid accumulation and increased lipid productivity in *Thalassiosira weissflogii*, while in *Chaetocheros mulleri,* nitrogen limitation strongly induced lipid accumulation, while phosphorus limitation did not induce higher lipid production. Like this study, silicon starvation had a very modest or no effect on the total lipid content in these marine microalgae [56]. On the contrary, under silica, deficiency increased total fatty acid content in *Navicula* sp. and *Amphora* sp. [57]. Lipid content in *Nitzschia* sp. S5 in this work is comparable to literature values for the same strain [58].

Total chlorophyll content was measured using a simple spectrophotometric method. As expected, nitrogen limitation had the strongest impact on total chlorophyll content compared to silicon and phosphorus. Nitrogen is a structural component of chlorophyll molecules; therefore, nitrogen stress might decrease light harvesting and photosystem II activity efficiency, resulting in reduced photosynthetic rates [40,59]. Relatively high chlorophyll content of 2.31% was obtained in biomass grown in the absence of a phosphorus source. Due to phosphate reserve in the cell, the effect of phosphorus limitation was not observed. The lack of intracellular phosphorus is connected to the cell’s inability to produce ATP and NADPH needed for chlorophyll synthesis in the absence of phosphorus supply [55]. However, the highest total chlorophyll content was obtained under phosphorus-replete conditions, while silicon showed no significance. A similar content of total chlorophyll was in accordance with literature data for diatoms [44,60,61].

The most abundant fatty acids in *Nitzschia* sp. S5, under all studied conditions, were palmitoleic (C16:1, *cis* 9), followed by palmitic (C16:0) and myristic (C14:0) acids (Table 2). These fatty acids comprised more than 90% of total fatty acids, similar to other *Nitzschia* strains [62,63]. The remaining fatty acids in cell biomass comprised C 17 (C17:0, C17:1), C18 (C18:0, C18:1, C18:2), and long-chain (C20:5, C24:0 and C24:1, *cis* 15) fatty acids. However, the profile of remaining fatty acids differed from that reported for other *Nitzschia* strains [62,64]. Although all studied macronutrients affected the fatty acid composition of cell lipids, the effect of phosphorus and silicon concentrations was more significant. The content of palmitoleic acid consequently resulted in a high content of monounsaturated fatty acids (MUFA), comprising more than 50% of total lipids under all studied conditions. Similar findings were reported by Qiao et al. and Sahin et al. for nitrogen limitation, where the content of monounsaturated but also saturated fatty acids (SFA) increased under nitrogen-deficient conditions [65,66]. Along with palmitic acid (C16:0), myristic acid (C14:0) comprised most of the saturated fatty acids, while the most abundant polyunsaturated fatty acid was eicosapentaenoic acid (C20:5, *cis* 5, 8, 11, 14, 17). The content of the least abundant class of fatty acids, polyunsaturated fatty acids (PUFA), was not significantly affected by the concentration of these three macronutrients. The highest content of PUFA was obtained under phosphorus limitations and nitrogen-repleted conditions. Similarly, Lin et al. observed a decrease in PUFA under nitrogen limitation, while silicon and phosphate limitation had the opposite effect [56]. Most diatoms have a high content of very long chain polyunsaturated fatty acids (VLC-PUFAs), such as EPA and ARA (arachidonic acid; C20:4, n-6) [64,67,68]. The highest contents of EPA in this study were obtained at nitrogen concentration of 6 and 8 mM and 0 mM of phosphorus (3.19%, 3.51%, and 3.72%, respectively), while the higher silicon concentrations had a strong negative effect on EPA content, resulting in only 0.37% at 2 mM (Figure 1). Several studies on lipid synthesis showed that nutrient starvation increases EPA and DHA content in diatoms, but in our study, this was observed only for nitrogen and phosphorus but not silicon starvation [69,70,71].

### 2.2. Effect of Nitrogen, Silicon, and Phosphorus Molar Ratios on Growth and Composition of Biomass

**Figure 2:**
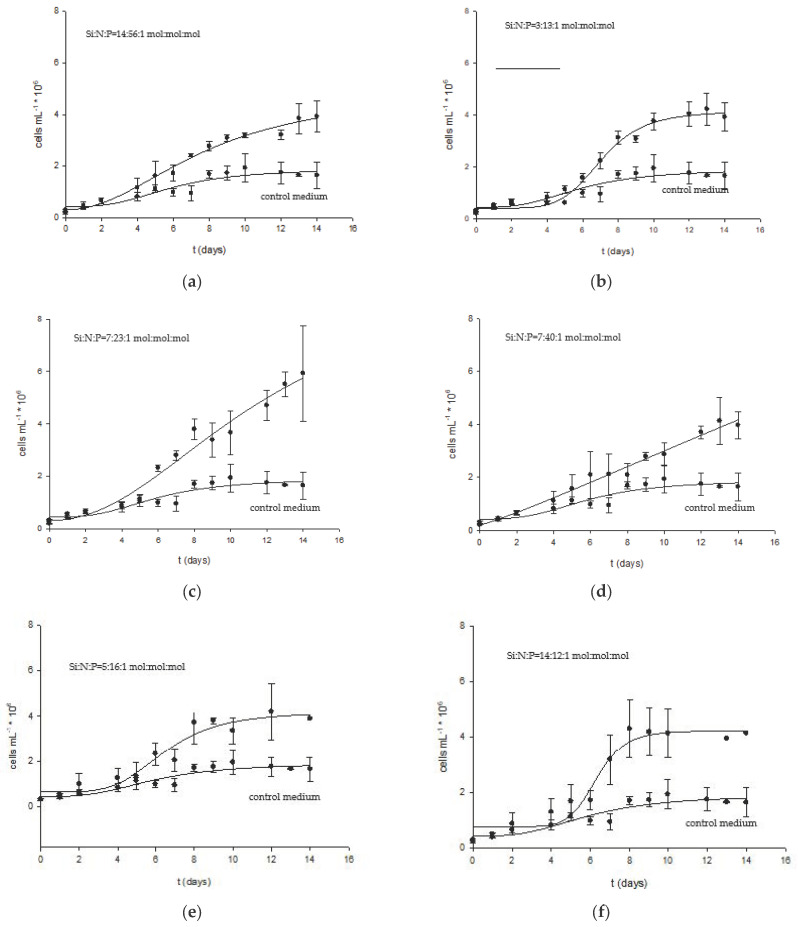
The growth curves of *Nitzschia* sp. in original (control medium) and modified f/2 media with different Si:N:P ratios: (**a**) Si:N:P = 14:56:1 mol:mol:mol; (**b**) Si:N:P = 3:13:1 mol:mol:mol; (**c**) Si:N:P = 7:23:1 mol:mol:mol; (**d**) Si:N:P = 7:40:1 mol:mol:mol; (**e**) Si:N:P = 5:16:1 mol:mol:mol; and (**f**) Si:N:P = 4:12:1 mol:mol:mol.

Previous experiments showed that the growth and biomass composition of *Nitzschia* sp. S5 is greatly influenced by each of the three studied nutrients. The concentration of remaining macronutrients in the growth medium at the end of cultivation suggests that diatom growth was limited by more than macronutrients in the later cultivation phase (Supplementary Materials, Table S1). Phosphate was completely used under all cultivation conditions except in culture broth with the highest initial phosphate concentration. Concentrations of silicon and nitrogen were below the limiting concentration reported in the literature for diatoms [2,39,59,72,73,74]. Therefore, in continuation of this study, the combined effect of nitrogen, silicon, and phosphate on diatom biomass growth and biochemical composition was further investigated. The concentrations of macronutrients were chosen based on the value of specific rates obtained in the previous experiment (Table 1). Although phosphorus concentrations above 0.036 mM inhibited the growth, higher phosphate was also tested to avoid possible growth limitation by this element in the later phase of cultivation in a modified Guillard f/2 medium with high concentrations of silicon and nitrogen. Six modified growth media with optimised concentrations of macronutrients used in the second phase of the experiment are presented in 3. Materials and Methods, 3.3. Optimisation of Growth Medium for High Biomass Productivity. The control culture was grown on the original Guillard f/2 medium. The growth curves of *Nitzschia* sp. S5 had a characteristic shape for single-cell organisms. The length of each growth phase depended on the composition of the growth medium. Thus, the longest exponential phase was observed in cultures grown at higher nitrogen phosphor ratios of 56:1, 23:1, and 40:1 (Figure 2, Table 3).

According to the Redfield nitrogen-to-phosphorus molar ratio, the values of N:P below 16:1 mol:mol suggest that the diatom was limited by phosphorus, which could cause the prolongation of the lag phase [75]. Despite the unfavourable ratio of macronutrients in the growth medium, the specific growth increased compared to the control culture. The cell number and dry weight concentration increased in cultures grown on six modified f/2 media compared to the control culture grown in the original Guillard f/2 growth medium. Dry weight concentrations at the cultivation end were significantly higher than in the control culture (from 0.51 to 0.73 g L^−1^ versus 0.28 g L^−1^). The highest biomass concentration and biomass productivity (0.73 g L^−1^ and 52.04 mg L^−1^ day^−1^, respectively) were obtained at a N:Si:P ratio of 7:23:1. This value differs from Redfield’s ratio (Si:N:P = 15:16:1 mol:mol:mol), generally accepted for diatoms, but deviations from this ratio are reported in the literature for the same diatom strains, especially when culture media composition or growth conditions significantly vary [20,76,77]. Usually, it is used as an indicator of nutrient limitation (Si:N:P molar ratio below 15:16:1 mol:mol:mol) [78]. The highest growth rate of 0.29 h^−1^ was obtained at the molar ratio Si:N:P = 3:13:1 mol:mol:mol, which was slightly lower than the maximal value (0.35 h^−1^) obtained in the previous experiment at the molar ratio of Si:N:P = 28:24:1 mol:mol:mol, which was very close to the optimal ratio for diatoms [76]. The specific optimal macronutrient ratios for each of the five strains of *Navicula* and *Nitzschia* reported in the study of Yang et al. suggest that the composition of these nutrients in a growth medium is strain-dependent [20].

Over 90% of the silicon and phosphate were used in cultures grown in all modified growth media, while the consumption of nitrate varied (Supplementary Materials, Table S1). Higher nutrient loading of phosphate (growth medium with Si:N:P ratios of 5:16:1 and 4:12:1 mol:mol:mol) did not result in complete silicon or nitrate consumption. However, increased silicon concentration (from 0.5 to 1 mM) improved nitrogen consumption. The most efficient utilisation of all three nutrients was observed in a modified medium with a molar ratio of Si:N:P = 7:23:1 mol:mol:mol.

As mentioned, nutrient limitation leads to the accumulation of storage products, meaning carbohydrates and lipids. So, the decreasing trend for lipid and carbohydrate content with more nutrient supply is expected. Carbohydrate content was the highest in diatom cells grown in the control medium (19.73%) and decreased to 3.11 and 4.19% at molar ratios Si:N:P = 5:16:1 and 4:12:1 mol:mol:mol, respectively. On the other hand, protein content increased from 15.34% at the control to a maximum of 21.48% and 21.00% at Si:N:P ratios of 3:13:1 and 7:23:1, respectively. The diatom composition was similar to literature data for different *Nitzschia* strains and other diatoms [2,47,74,79,80]. In exponentially growing cells, protein content almost exceeds carbohydrate content, which, in turn, usually exceeds the lipid content [81]. Lipid and carbohydrate contents are usually lower in actively growing cells than in senescent cells, while the protein content tends to remain constant and decreases with cell senescence [82,83,84]. In actively growing cells, protein content ranges from 17 to 35%, carbohydrates from 4 to 20% and lipids from 2 to 7% of cell dry weight [85]. The values obtained for cell biomass grown in six modified growth media (Table 4) are within those ranges. Since this experiment aimed to achieve high biomass concentration, a Si:N:P ratio of 7:23:1 mol:mol:mol was further used to cultivate *Nitzschia* sp. S5.

The fatty acid profile of diatom biomass (Table 4) was not significantly different from biomass grown at different molar ratios of main macronutrients (Figure 1). Fatty acids composition was similar to other *Nitzschia* strains comprising palmitoleic, myristic, palmitic, and eicosapentaenoic acids [3,7,58,86,87]. Furthermore, margaric (C17:0) and heptadecanoic (C17:1) acids were also observed in total cell lipids, suggesting possible bacterial contamination of microalgal culture [63]. The increase in eicosapentaenoic fatty acid (EPA; C20:5) content in the nutrient-rich growth medium indicates that lower molar Si:N:P ratios of 4:12:1 and 5:16:1 mol:mol:mol favour synthesis of this long-chain unsaturated fatty acid. The highest EPA content of 9.42% and 9.31 was observed at the Si:N:P ratios of 4:12:1 and 5:16:1 mol:mol:mol, respectively.

### 2.3. Fed-Batch Cultivation

**Figure 3:**
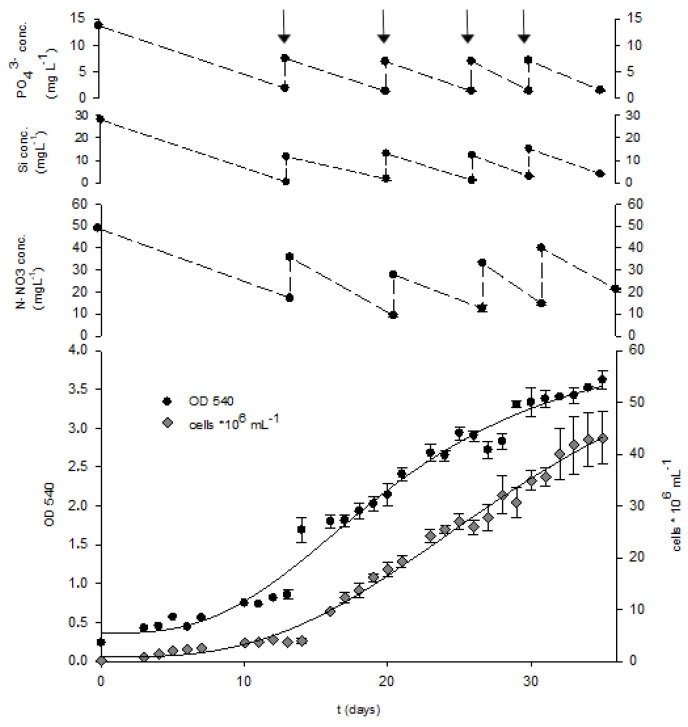
The growth curves of *Nitzschia* sp. S5: cell concentration (cells mL^−1^) and optical density (OD 540 nm) during the fed-batch cultivation and macronutrient concentrations. The culture was fed on the 13th, 20th, 26th, and 30th day of cultivation (arrows).

The low solubility of silicon (1.5–1.7 mM) in culture medium is one of the main obstacles to achieving high densities of microalgal cells, especially in growth media with high concentrations of other nutrients. Feeding the microalgal culture with a concentrated solution of macronutrients and keeping their concentration of nutrients below inhibition concentration enables the further increase in cell yield. The fed-batch strategy is commonly used in microbial fermentation to avoid substrate inhibition and achieve high cell/product concentrations under controlled dynamics of the addition of concentrated substrate/s solution, such as in the production of bioethanol, microbial lipids, lactic acid, etc. [88,89,90]. Initial nutrient concentration in the medium was equal to the optimal ratio Si:N:P = 7:23:1 mol:mol:mol. For the first thirteen days, *Nitzschia* sp. S5 was cultivated in batch mode until the late exponential phase when silicon was almost depleted from culture broth (0.42 mg L^−1^ Figure 3). Fed-batch cultivation was started by adding a concentrated nutrient solution containing silicon, nitrogen, and phosphate sources at optimal molar ratio. The amount of added nutrients provided an approximate increase of 0.3 g L^−1^ of biomass estimated from data from previous experiments (2.2. Effect of Nitrogen, Silicon, and Phosphorus Molar Ratios on Growth and Composition of Biomass).

The culture growth was continuously monitored by measuring optical density and cell concentration (Figure 3). When the growth rate decline was observed, the culture was fed with a concentrated nutrient solution. The culture was fed four times, on the 13th, 20th, 26th, and 30th days of cultivation. The gradual addition of nutrients more than doubled the cell dry weight concentration (2.23-fold) compared to batch cultivation (first phase of cultivation) in the optimised medium and was 5.82-fold higher compared to the control culture grown on the original f/2 medium. The growth rate steadily decreased during the cultivation from 0.13 day^−1^ after the first nutrient addition to 0.08 day^−1^ at the end of cultivation (Table 5). A decrease in growth rate indicates self-shading due to the high biomass concentration, especially in the later growth phase [5].

The productivity in batch cultivation was higher (60.71 mg L^−1^ day^−1^) compared to fad-batch cultivation (Table 5). The difference in biomass productivity during fed-batch cultivation was not statistically significant (45.41–49.14 mg L^−1^ day^−1^). The performance of the diatom during the two growth modes suggests the culture was limited by another factor, possible light at a late phase of growth due to the self-shading of the cells. Due to the characteristics of different diatom species, cultivation modes, the composition of cultivation media, and feeding strategy, the results are difficult to compare with data reported in the literature. Fed-batch cultivation enables high biomass yield of different types of microorganisms as well as in diatom cultivations. In fed-batch cultivation of *Isochrysis galbana* aerated with CO_2_-enriched air and fed with a nitrogen source, the final biomass concentration was 1.88 g L^−1^, while the biomass concentration in batch culture was 0.42 g L^−1^ [5]. Ozkan and Rorrer reported ten times higher cell number density with applied multistage nutrient feeding compared to the two-stage Si addition process [21]. This study’s analysis of three macronutrients showed that silicon was first depleted from the culture broth, causing a decrease in diatom growth (Figure 3).

Furthermore, phosphorus concentration was relatively low before feeding time, especially before the first and second feedings. On the contrary, nitrogen source concentration was above the limiting concentration during the cultivation and, therefore, did not affect the growth. Since the previous experiments showed that nitrogen limitation stimulates the accumulation of storage compounds in *Nitzschia* sp. S5 more efficiently than silicon deprivation, the low content of lipids in biomass was expected, and analysis of biomass confirmed an assumption (from 1.94 to 2.42%; Table 5). Low lipid content and productivity observed during batch and fed-batch cultivation (1.18 and 1.28 mg L^−1^ day^−1^) suggest *Nitzschia* sp. S5 might not be suitable for biodiesel production. However, biomass yield as well as lipid content during phototrophic cultivation could be improved by increasing carbon dioxide concentration [91]. The content of proteins in biomass indicates that cells were actively growing without accumulation of energy reserve. The content of carbohydrates moderately increased during fed-batch cultivation, especially after the first feeding (from 8.04 to 14.50%, respectively), with the final 12.92%.

Fatty acids analysis (Table 6) showed a typical profile with the predominant palmitoleic, myristic, palmitic, and eicosapentaenoic [3,7,58,86,92]. Also, odd-chain fatty acids, margaric (C17:0) and heptadecanoic (C17:1), were detected. These fatty acids are also used as biomarkers for bacterial contamination of microalgae cultures [63]. However, they can also be found in axenic microalgal cultures, and their content in this study was low, similar to data published in the literature [87,93]. The content of polyunsaturated eicosapentaenoic acid significantly increased compared to previous experiments, especially in the early exponential phase. This fatty acid is distributed in all lipid classes, and its content in total lipids strongly depends on the growth phase, cultivation conditions and microalgal strain [64,66]. Since the culture was in the growth phase, eicosapentaenoic acid was used as a building block for the membrane lipids—phospholipids and glycolipids, and less for neutral lipids—mainly for energy storage. Arachidonic acid was detected, but its content in biomass was considerably lower than eicosapentaenoic acid. The content of arachidonic acid was increased from the middle to late exponential phase in accordance with published studies [66]. Several unsaturated fatty acids (C18:2 and C20:4) were also accumulated in biomass during the later exponential phase (fed-batch cultivation), increasing overall unsaturated fatty acid content compared to the early phase of cultivation (batch cultivation) similar to *Nitzschia closterium* MACC/B222 [94].

## 3. Materials and Methods

### 3.1. Microalgae

The microalga *Nitzschia* sp. S5 was acquired from the microalgae culture collection of the Laboratory for Biochemical Engineering, Industrial Microbiology, and Malting and Brewing Technology at the Faculty of Food Technology and Biotechnology [13]. Culture was maintained by subculturing using original Guillard f/2 medium and grown at room temperature (approx. 20 °C) under warm white light lamps with a luminous intensity of 3000 lux and light–dark photoperiod of 16:8 (h h^−1^).

### 3.2. Growth Media Preparation and Cultivation Conditions

*Nitzschia* sp. S5 was cultivated in a Guillard f/2 medium with the addition of sodium metasilicate as a source of silicon [13]. The following chemicals were used for growth medium preparation: NaNO_3_ (Kemika; Zagreb, Croatia), NaH_2_PO_4_ · H_2_O (Kemika; Zagreb, Croatia), Na_2_SiO_3_ · 9H_2_O, FeCl_3_ 6H_2_O (Sigma Aldrich; St. Louis, MI, USA), Na_2_EDTA · 6H_2_O (Carlo Erba; Val-de-Reuil, France), MnCl_2_ · 4H_2_O (Kemika; Zagreb, Croatia), ZnSO_4_ · 7H_2_O (Kemika; Zagreb, Croatia), CoCl_2_ · 6H_2_O (Kemika; Zagreb, Croatia), CuSO_4_ · 5H_2_O (Kemika; Zagreb, Croatia), Na_2_MoO_4_ · 2H_2_O (Kemika; Zagreb, Croatia), thiamine (Acros Organics; Belgium), biotin (Sigma Aldrich; St. Louis, MI, USA), cyanocobalamin (Sigma Aldrich; St. Louis, MI, USA).

The inoculum was prepared by gradually increasing culture volume by subculturing weekly, starting from 20 mL and going up to 250 mL of growth medium. Inoculum concentration was 10% (*v*/*v*) of the culture volume. Diatom was grown on a rotary shaker at 200 rpm, under warm white light lamps, light-to-dark photoperiod 16:8 h, and 23 ℃. Batch cultures were grown until the cells entered the stationary phased growth.

### 3.3. Optimisation of Growth Medium for High Biomass Productivity

Optimisation of the growth medium was conducted in three consecutive steps. *Nitzschia* sp. S5 f/2 was cultivated in the Guillard f/2 medium. First, the effect of nitrogen, silicon, and phosphorus concentrations on the growth of diatom was studied separately under the conditions described above (Section 3.2). The following concentrations of the macronutrients were investigated:

- Nitrogen: 0 mM (negative control), 0.88 mM (concentration in original f/2 medium), 2 mM, 3.5 mM, 6 mM, and 8 mM;
- Silicon: 0 mM (negative control), 0.1 mM (concentration in original f/2 medium), 0.5 mM, 1 mM, 1.5 mM, and 2 mM;
- Phosphorus: 0 mM (negative control), 0.1 mM (concentration in original f/2 medium), 0.5 mM, 1 mM, 1.5 mM, and 2 mM.

All experiments were conducted in 500 mL borosilicate Erlenmeyer flasks with 250 mL of growth medium and 10% (*v/v*) inoculum. Additional control cultivation with 0 mM silicon was conducted in plastic Erlenmeyer flask to estimate the effect of silicon leaching from borosilicate Erlenmeyer flasks.

Next, the effect of silicate, nitrogen, and phosphate molar ratios on diatom growth was investigated. The molar ratios were chosen based on results from previous experiments. The composition of six modified Guillard f/2 media used for cultivations is presented in Table 7.

Finally, fed-batch cultivation was conducted using the growth medium with an optimised micronutrients ratio with Si:N:P = 6.67:40:1 mol:mol:mol. The growth conditions of microorganism were as mentioned above (Section 3.2). All experiments were carried out in triplicates and conducted under the conditions described above. The batch cultivation lasted until the late exponential growth phase, when the growth rate slowed, and macronutrients were almost depleted from the culture broth. Fed-batch cultivation was started by feeding the culture with a concentrated solution of macronutrient (Na_2_SiO_3_ · 9H_2_O, 30 g L^−1^; NaNO_3_, 75 g L^−1^; and NaH_2_PO_4_ · H_2_O, 5 g L^−1^). The volume of concentrated solution fed to culture broth provided an optimal ratio of the macronutrients (Si:N:P = 6.67:40:1 mol:mol:mol). The culture was fed four times.

During cultivation, biomass concentration (number concentration and optical density), concentrations of silicon, nitrogen and phosphorus, biomass composition, and fatty acid composition were determined. Based on the obtained data, specific growth rates and productivities were calculated.

### 3.4. Growth Parameters

Microalgae growth was monitored by daily measurement of culture optical density at 540 nm (OD540) (Cary 100 UV-Vis spectrophotometer, Agilent; Santa Clara, CA, USA). Cell concentration was measured using the Thoma chamber and light microscope (Olympus, model CH20; Tokyo, Japan).

At the end of cultivation, microalgae biomass was collected by centrifugation at 8000 rpm for 15 min at 4 °C (ThermoScientific, SL 8R Cell Culture Centrifuge; Waltham, MA USA). Biomass was washed at least two times with distilled water [95], and transferred in a dry and weighed glass tube. Biomass was dried by lyophilisation, weighed, and stored at room temperature in hermetically closed glass tubes until further analysis. Biomass concentration was determined gravimetrically and calculated as follows:

(1) $$X=\frac{m_{1}-m_{0}}{V\;}(g\;L^{-1})$$

where *m*_1_ stands for the weight of biomass and glass tube after lyophilisation (g), *m*_0_ stands for a mass of empty glass tube (g), and *V* stand for a volume of culture (L).

### 3.5. Determination of Silicate, Nitrate, and Phosphate Concentration

The concentration of remaining silicon, nitrogen, and phosphorus in culture supernatants were determined using Spectroquant^®^ Merck test kits (Silicic Acid Test (Ca. No. 101813), Nitrate Test (Ca. No. 109713), and Phosphate Test (Ca. No. 114848), respectively). Concentrations of macronutrients were calculated using a calibration line ranging from 1 to 25 mg N-NO_3_ L^−1^ of sodium nitrate, 0.1 to 1.5 mg Si L^−1^ of sodium metasilicate, and 0.05 to 3.5 mg PO_4_^3−^ L^−1^ of disodium phosphate.

### 3.6. Biomass Composition

Lyophilised biomass was used for the analysis of biomass composition. All analyses were carried out in triplicates, and results are presented as average value ± standard deviation.

#### 3.6.1. Carbohydrate Content

Total carbohydrates in microalgae biomass were determined using the method developed by the National Renewable Energy Laboratory [96]. After two-step sulfuric acid hydrolysis of carbohydrates, monosaccharides were then quantified using an ultraperformance liquid chromatograph (Agilent. 1290 Infinity II; Santa Clara, CA, USA), equipped with a refractive index detector (Agilent, Infinity II 1260; Santa Clara, CA, USA) and ion-exclusion HPLC column Rezex ROA, Organic Acid H+ (8%) (Phenomenex, Torrance, CA, USA). The concentration of analytes was determined using calibration lines. The calibration lines for standard solutions of analytes are presented in Table 8.

#### 3.6.2. Total Protein Content

For total protein content determination, 1–1.3 mg biomass was resuspended in 1 mL of 1 M NaOH (Merck, Darmstadt, Germany) and incubated in Thermo-shaker (BioSan TS-100; Riga, Latvia) for 20 min at 100 °C and 3000 rpm. The alkali biomass hydrolysate was cooled to room temperature, and total proteins were determined according to the Lowry method [13].

#### 3.6.3. Lipid Content and Fatty Acid Composition

Total lipids were determined as total fatty acid methyl esters (FAME) by in situ transesterification after the protocol for analysis of microalgal biomass developed by the National Renewable Energy Laboratory [97]. The FAME content was determined using a gas chromatography (Shimadzu GC-2010 Plus Capillary gas chromatograph; Kyoto, Japan) equipped with a flame ionisation detector (FID) and high-cyanopropyl capillary column (ZB-FAME; 30 m × 0.25 mm × 0.2 µm, Phenomenex; Torrance, CA, USA). Samples were injected in split mode (split ratio 1:15) with an AOC-20i injector. Helium was used as a carrier gas. The injector and detector temperatures were 250 and 260 °C, respectively. The temperature programme was 100 °C for 2 min, increase in temperature from 100 to 140 °C at a rate of 3 °C min^−1^, followed by temperature increase from 140 °C to 190 °C at a rate of 3 °C min^−1^, then up to 260 °C at a rate of 30 °C min^−1^ and holding at that temperature for 2 min. Shimadzu LabStation Software (version 1.0.5809.35295) was used for instrument control, data acquisition. and data analysis (integration, retention times and peak areas). FAMEs were identified and quantified using retention time and calibration lines of FAME standard solution (Supelco FAME Mix, C4-C24. Catalog No.: 18919-1AMP, Bellefonte, PE, USA). The content of identified FAME in total FAMEs was calculated.

#### 3.6.4. Pigment Content

Chlorophyll a and c were determined using a spectrophotometric method described by Jeffrey and Humphrey [98]. Acetone (90% *v*/*v*) was used as an extraction solvent. Ten millilitres of culture was centrifuged, and biomass was washed twice with deionised water. The supernatant was discarded and 1 g of glass beads and 2 mL of 90% (*v/v*) were added, vigorously vortexed for 1 min, and then cooled down for 2 min on ice. This step was repeated two times. The supernatant was separated by centrifugation, transferred into a clean tube, and kept on ice in the dark. Extraction was repeated until biomass was colourless. After each extraction step, supernatants were combined. The absorbance was measured at 630, 647, 664, and 750 nm (Equations (2) and (3)) using a spectrophotometer. The pigment content was calculated using the following empirical equation [98]:

(2) $$\mathrm{Chl}\;a=\frac{11.85\times \left({OD}_{664}-{OD}_{750}\right)-1.54\times \left({OD}_{647}-{OD}_{750}\right)-0.08\times \left({OD}_{630}-{OD}_{750}\right)\times V_{e}}{L\times V_{f}}$$

(3) $$\mathrm{Chl}\;c=\frac{-1.67\times \left({OD}_{664}-{OD}_{750}\right)-7.6\left({OD}_{647}-{OD}_{750}\right)+24.52\times \left({OD}_{630}-{OD}_{750}\right)\times V_{e}}{L\times V_{f}}$$

where V_e_ is the total volume of extract (mL), V_f_ is the volume of culture used for analysis (L), and L cuvette path length (cm).

### 3.7. Bioprocess Efficiency Parameters

The biomass (Pr_x_) and lipid (Pr_L_) productivities were calculated at the end of cultivation according to the following equations:

(4) $${\mathrm{Pr}}_{X}=\frac{X-X_{0}}{t-t_{0}}$$

(5) $${\mathrm{Pr}}_{L}=\frac{C_{L}-C_{L0}}{t-t_{0}}$$

X_0_ and X are the biomass concentrations at the beginning of cultivation (t_0_) and at a specific time (t), respectively; C_L0_ and C_L_ are concentrations of lipids at the beginning of cultivation (t_0_) and at a specific time (t), respectively.

Specific growth rate (μ) was determined as a first-order reaction. The equation for exponential growth was linearised:

ln N = ln N_0_ + µ·t (6)

where μ was determined from the slope of the linearised regression line.

### 3.8. Statistical Analysis

Data were processed with Excel (version 2010; Microsoft Corp.; Redlands, WA, USA), SigmaPlot (version 11; SYSTAT Software, Inc.; San Jose, CA, USA). In each case, means ± standard error for n number of samples are given.

The statistical significance of differences was determined using one-way ANOVA followed by multiple comparison tests (Turkey’s test). A significance level of 95% (*p* < 0.05) was accepted.

## 4. Conclusions

In this study, optimisation of growth medium composition for enhanced biomass production of diatom *Nitzschia* sp. S5 was conducted. The effect of the concentration of nitrogen, silicon, and phosphorus was first investigated separately for each macronutrient and afterwards by changing their molar ratio in the growth medium. The maximum dry weight concentration of 0.49 g L^−1^ was obtained in a growth medium with 1 mM of silicon. Si:N:P molar ratio of 7:23:1 mol:mol:mol resulted in the highest biomass concentration of 0.73 g L^−1^, which is 2.64-fold higher than the biomass concentration obtained in the control Guillard f/2 medium. A fed-batch cultivation with optimised growth medium composition was applied to improve cell biomass production further. The concentration of diatom biomass at the end of cultivation was 1.63 g L^−1^, which is 5.82 times higher than the cell concentration obtained in the original Guillard (f/2) medium. Proteins were the most abundant macromolecule in cell biomass (11.41% to 64.10%). The highly valuable polyunsaturated eicosapentaenoic acid comprised 10.50% of total lipid at the end of fed-batch cultivation.

Under specific growth conditions, diatoms have the ability to accumulate lipids and different high-value compounds such as pigments, lipids, and antimicrobial substances. Therefore, diatoms could be considered an alternative feedstock for the production of biofuels in microalgal biorefinery in which additional revenue could be obtained from high-value by-products. Low biomass concentration and high production costs are the major obstacles to the commercialisation of microalgal biofuels. Improvement of cultivation strategy, including optimisation of cultivation conditions, bioprocess operation modes, and growth media composition, are crucial steps toward establishing microalgal biorefineries. In this study, a strategy for the enhancement of diatom biomass concentration was successfully established. Further research should focus on adjusting cultivation strategies toward accumulating lipids and other high-value products in microalgal biomass.

## Figures and Tables

**Table 1:** The effect of nitrogen, silicon, and phosphorus molar concentration (C) on cell growth during batch cultivation in f/2 growth medium: dry cell weight concentration (X), specific growth rate (μ), biomass (Pr_x_), and lipid productivity (Pr_L_) at the end of batch cultivation.

Nutrient	C (mM)	Si:N:P (mol:mol:mol)	X (g L^−1^)	µ (day ^−1^)	Pr_x_ (mg L^−1^day^−1^)	Pr_L_ (mg L^−1^day^−1^)
N	0	3:0:1	0.10 ± 0.01	0.22 ± 0.01	8.53 ± 0.99	1.26 ± 0.14
	0.88	3:24:1	0.20 ± 0.04	0.24 ± 0.03	16.87 ± 2.98	2.18 ± 0.39
	2	56:3::1	0.24 ± 0.01	0.26 ± 0.02	20.02 ± 0.78	2.14 ± 0.08
	3.5	97:3:1	0.23 ± 0.04	0.32 ± 0.04	19.31 ± 3.26	1.86 ± 0.31
	6	167:3:1	0.23 ± 0.03	0.29 ± 0.01	19.05 ± 0.22	1.51 ± 0.02
	8	222:3:1	0.13 ± 0.06	0.21 ± 0.002	19.79 ± 4.84	2.36 ± 0.58
Si	0 *	0:24:1	0.05 ± 0.07	0.04 ± 0.02	3.85 ± 0.77	/*
	0	0:24:1	0.09 ± 0.048	0.26 ± 0.009	7.97 ± 0.73	0.69 ± 0.06
	0.1	3:24:1	0.31 ± 0.04	0.25 ± 0.011	23.34 ± 1.06	2.13 ± 0.09
	0.5	14:24:1	0.38 ± 0.08	0.22 ± 0.02	29.51 ± 2.08	4.12 ± 0.29
	1	28:24:1	0.49 ± 0.09	0.35 ± 0.02	37.61 ± 2.92	2.13 ± 0.17
	1.5	42:24:1	0.26 ± 0.08	0.26 ± 0.01	20.11 ± 1.62	0.46 ± 0.04
	2	56:24:1	0.27 ± 0.02	0.19 ± 0.04	20.8 ± 0.58	0.29 ± 0.01
P	0	-	0.101 ± 0.04	0.24 ± 0.03	8.43 ± 0.69	1.56 ± 0.27
	0.036	3:24:1	0.34 ± 0.06	0.25 ± 0.06	28.50 ± 1.56	4.01 ± 0.38
	0.15	0.7:6:1	0.27 ± 0.09	0.10 ± 0.02	22.7 ± 2.00	2.08 ± 0.03
	0.36	0.3:2.4:1	0.25 ± 0.02	0.12 ± 0.01	20.85 ± 0.43	2.59 ± 0.18

* cultivation was conducted in plastic Erlenmeyer flasks.

**Table 2:** Effect of nitrogen, silicon, and phosphorus concentration on fatty acid composition.

Fatty Acid	Nitrogen (mM)					
(%)	0	0.88	2	3.5	6	8
C14:0	10.76 ± 0.49	11.91 ± 0.22	12.41 ± 0.47	10.29 ± 0.21	9.40 ± 0.21	9.22 ± 0.15
C16:0	28.75 ± 0.55	27.03 ± 0.75	28.00 ± 0.03	33.25 ± 0.58	36.58 ± 0.52	32.22 ± 0.98
C16:1, *cis* 9	57.22 ± 0.95	56.09 ± 0.46	56.88 ± 1.34	53.16 ± 0.49	52.33 ± 0.23	53.06 ± 0.14
C17:1, *cis* 10	0.96 ± 0.45	1.05 ± 0.29	1.02 ± 0.37	1.45 ± 0.34	0.42 ± 0.05	1.21 ± 0.37
C20:5, *cis* 5,8,11,14,17	2.39 ± 1.08	2.92 ± 0.81	2.72 ± 1.62	2.28 ± 0.74	3.19 ± 0.66	3.51 ± 0.44
C24:0	n.d.	0.53 ± 0.13	n.d.	n.d.	1.59 ± 0.18	n.d.
C24:1, *cis* 15	n.d.	0.28 ± 0.07	n.d.	1.68 ± 0.28	n.d.	n.d.
SFA	39.76 ± 0.58	39.79 ± 1.11	40.63 ± 0.59	43.80 ± 0.81	46.21 ± 0.65	42.21 ± 0.67
MUFA	57.86 ± 0.50	57.21 ± 0.58	57.55 ± 1.02	53.93 ± 0.46	52.37 ± 0.21	54.27 ± 0.23
PUFA	2.39 ± 1.08	2.99 ± 0.77	1.81 ± 1.62	2.28 ± 0.74	1.42 ± 0.66	3.51 ± 0.44
**Fatty Acid**	**Silicon (mM)**					
**(%)**	**0**	**0.1**	**0.5**	**1.0**	**1.5**	**2.0**
C14:0	12.46 ± 1.18	12.88 ± 0.99	9.92 ± 0.60	10.35 ± 0.28	12.79 ± 0.25	12.50 ± 0.93
C16:0	26.73 ± 2.43	25.45 ± 1.11	30.94 ± 2.89	24.50 ± 1.03	16.10 ± 0.23	8.94 ± 2.75
C16:1, *cis* 9	53.49 ± 3.22	57.11 ± 0.66	54.96 ± 1.42	60.35 ± 1.02	65.20 ± 0.43	66.75 ± 4.78
C17:0	1.03 ± 0.65	0.69 ± 0.27	0.04 ± 0.04	2.91 ± 0.42	4.18 ± 0.43	6.24 ± 4.26
C17:1, *cis* 10	0.29 ± 0.19	1.67 ± 0.85	0.98 ± 0.50	0.05 ± 0.05	n.d.	n.d.
C18:0	0.46 ± 0.26	n.d.	1.10 ± 1.10	0.28 ± 0.27	n.d.	n.d.
C18:1, *cis* 9	n.d.	n.d.	n.d.	n.d.	n.d.	n.d.
C18:2, *cis* 9,12	1.26 ± 0.77	0.48 ± 0.24	0.55 ± 0.30	0.10 ± 0.06	n.d.	0.34 ± 0.33
C20:5, *cis* 5,8,11,14,17	1.45 ± 0.90	0.82 ± 0.42	0.74 ± 0.37	n.d.	n.d.	0.37 ± 0.37
C24:0	n.d.	0.15 ± 0.09	0.05 ± 0.03	0.74 ± 0.69	0.36 ± 0.31	2.74 ± 2.33
C24:1, *cis* 15	n.d.	n.d.	n.d.	n.d.	n.d.	n.d.
SFA	41.21 ± 2.19	39.45 ± 1.63	42.64 ± 2.60	39.09 ± 0.93	34.33 ± 0.44	30.41 ± 3.58
MUFA	55.99 ± 3.15	59.24 ± 1.10	56.07 ± 1.99	60.81 ± 0.95	65.32 ± 0.45	68.42 ± 4.09
PUFA	2.79 ± 1.69	1.30 ± 0.66	1.29 ± 0.65	0.09 ± 0.06	0.36 ± 0.35	1.17 ± 0.89
**Fatty Acid**	**Phosphate (mM)**					
**(%)**	**0**	**0.036 mM**	**0.15 mM**		**0.36 mM**	
C14:0	9.10 ± 1.34	10.41 ± 0.85	11.81 ± 0.08		11.44 ± 1.01	
C16:0	32.02 ± 4.13	24.57 ± 1.32	21.07 ± 0.69		22.98 ± 0.43	
C16:1, *cis* 9	53.27 ± 5.28	61.09 ± 1.95	63.15 ± 2.79		61.65 ± 1.05	
C17:1, *cis* 10	1.24 ± 0.36	1.41 ± 0.51	2.31 ± 1.32		2.68 ± 0.49	
C18:1, *cis* 9	n.d.	1.95 ± 1.95	n.d.		n.d.	
C20:5, *cis* 5,8,11,14,17	3.72 ± 0.43	1.26 ± 0.16	1.25 ± 0.71		1.71 ± 0.42	
SFA	41.64 ± 5.94	35.82 ± 1.48	33.69 ± 1.32		35.08 ± 0.91	
MUFA	54.64 ± 5.51	65.08 ± 0.49	66.26 ± 1.15		65.09 ± 1.07	
PUFA	3.72 ± 0.43	1.26 ± 0.16	1.25 ± 0.71		1.71 ± 0.42	

SFA—saturated fatty acids; MUFA—monounsaturated fatty acids; PUFA—polyunsaturated fatty acids; n.d.—not detected.

**Table 3:** Dry weight concentration (X), specific growth rate (µ), biomass and lipid productivity (Pr_x_, Pr_L_) at different nitrogen, silicon, and phosphate molar ratios in modified and original (control culture) Guillard f/2 growth media.

Si:N:P (mol:mol:mol)	X (g L^−1^)	Carbohydrates (%)	Proteins (%)	Total Lipids (%)	µ (day^−1^)	Pr_x_ (mg L^−1^ day^−1^)	Pr_L_ (mg L^−1^ day^−1^)
14:56:1 3:13:1 7:23:1 7:40:1 5:16:1 4:12:1 control	0.51 ± 0.06 0.56 ± 0.12 0.73 ± 0.07 0.66 ± 0.02 0.53 ± 0.10 0.51 ± 0.11 0.28 ± 0.14	11.53 7.08 8.04 9.58 3.11 4.19 19.73	19.14 ± 0.77 21.48 ± 1.10 21.00 ± 2.87 14.13 ± 1.00 18.25 ± 3.91 13.67 ± 2.57 15.34 ± 1.5	5.57 ± 0.57 5.20 ± 0.40 2.48 ± 0.19 2.27 ± 0.18 2.16 ± 0.24 1.99 ± 0.10 7.76 ± 0.32	0.18 ± 0.02 0.13 ± 0.001 0.14 ± 0.01 0.18 ± 0.02 0.18 ± 0.04 0.29 ± 0.07 0.15 ± 0.03	36.34 ± 5.31 39.86 ± 3.84 52.04 ± 2.52 45.15 ± 2.18 35.01 ± 3.55 38.67 ± 3.05 19.74 ± 3.25	2.02 ± 0.51 2.07 ± 0.35 1.29 ± 0.11 1.03 ± 0.09 0.76 ± 0.13 0.77 ± 0.11 1.02 ± 0.92

**Table 4:** The fatty acid composition of lipids from biomass is cultivated at different molar ratios of silicon, nitrogen, and phosphor sources in a growth medium.

Fatty Acid	Si:N:P (mol:mol:mol)						
(%)	Control	14:56:1	3:13:1	7:23:1	7:40:1	5:16:1	4:12:1
C14:0	10.60 ± 0.32	10.76 ± 0.42	14.40 ± 0.05	14.55 ± 0.79	14.97 ± 0.74	15.90 ± 0.35	16.04 ± 0.84
C14:1, *cis* 9	0.66 ± 0.02	0.89 ± 0.12	1.21 ± 0.02	1.65 ± 0.12	1.20 ± 0.19	0.88 ± 0.15	0.58 ± 0.17
C16:0	23.19 ± 0.26	17.84 ± 0.93	22.64 ± 0.19	15.98 ± 0.88	12.58 ± 1.99	10.75 ± 1.16	11.39 ± 3.58
C16:1, *cis* 9	56.12 ± 0.14	53.67 ± 1.45	53.81 ± 0.59	47.74 ± 0.61	54.41 ± 3.47	52.51 ± 3.73	49.14 ± 3.16
C17:0	1.06 ± 0.003	2.66 ± 0.423	2.93 ± 0.09	8.81 ± 1.17	8.30 ± 0.45	4.97 ± 1.08	3.45 ± 1.12
C17:1, *cis* 10	4.75 ± 0.15	7.76 ± 0.39	n.d.	3.02 ± 1.03	1.15 ± 0.58	5.68 ± 2.66	7.85 ± 2.31
C18:0	n.d.	n.d.	n.d.	n.d.	n.d.	n.d.	1.54 ± 1.54
C18:1, *cis* 9	n.d.	n.d.	n.d.	0.61 ± 0.38	n.d.	n.d.	1.84 ± 1.84
C20:5, *cis* 5, 8,11,14,17	3.48 ± 0.19	5.76 ± 1.07	3.97 ± 0.38	6.35 ± 0.79	5.20 ± 0.60	9.31 ± 2.88	9.42 ± 0.93
C24:0	n.d.	n.d.	n.d.	1.04 ± 0.80	1.92 ± 1.92	n.d.	0.26 ± 0.26
Total fatty acids	7.76 ± 0.32	5.57 ± 0.57	5.20 ± 0.40	2.48 ± 0.19	2.27 ± 0.18	2.16 ± 0.24	1.99 ± 0.10
SFA	34.84 ± 0.06	31.26 ± 0.59	40.16 ± 0.27	40.38 ± 0.61	37.77 ± 2.43	31.61 ± 2.46	32.68 ± 4.66
MUFA	61.68 ± 0.13	62.98 ± 1.07	55.87 ± 0.44	53.28 ± 1.04	57.03 ± 2.85	59.08 ± 1.70	57.90 ± 3.82
PUFA	3.48 ± 0.19	5.76 ± 1.07	3.97 ± 0.38	6.35 ± 0.79	5.19 ± 0.60	9.31 ± 2.88	9.42 ± 0.93

SFA—saturated fatty acids; MUFA—monounsaturated fatty acids; PUFA—polyunsaturated fatty acids; n.d.—not detected.

**Table 5:** Dry weight concentration (g L^−1^), specific growth rate (day^−1^), biomass and lipid productivity (mg L^−1^ day^−1^), and biomass composition (proteins, carbohydrates, total fatty acids, and total chlorophyll) during fed-batch cultivation.

Time (day)	X (g L^−1^)	µ (day^−1^)	Pr_x_ (mg L^−1^ day^−1^)	Pr_L_ (mg L^−1^ day^−1^)	Lipids (%)	Proteins (%)	Carbohydrates (%)
13	0.73 ± 0.04	0.12 ± 0.02	60.71 ± 4.41	1.28 ± 0.08	2.10 ± 0.06	11.41 ± 1.12	8.04 ± 0.85
20	1.03 ± 0.07	0.13 ± 0.01	49.14 ± 3.20	0.96 ± 0.06	1.94 ± 0.05	25.69 ± 1.85	14.50 ± 3.19
26	1.23 ± 0.04	0.10 ± 0.01	45.41 ± 1.46	1.05 ± 0.03	2.31 ± 0.17	28.64 ± 0.16	9.51 ± 0.79
30	1.46 ± 0.04	0.09 ± 0.03	48.57 ± 1.37	1.18 ± 0.03	2.42 ± 0.12	29.06 ± 0.15	12.92 ± 1.10
35	1.63 ± 0.05	0.08 ± 0.05	46.69 ± 0.57	1.06 ± 0.01	2.28 ± 0.11	28.05 ± 1.57	12.92 ± 1.07

**Table 6:** Fatty acid profile and total cellular lipids in microalgal biomass obtained using fed-batch cultivation.

Fatty Acid	Time (day)				
(%)	13	20	26	30	35
C14:0	19.50 ± 2.24	10.16 ± 3.22	11.48 ± 2.86	9.84 ± 3.09	2.91 ± 2.62
C14:1, *cis* 9	0.85 ± 0.02	1.39 ± 0.17	1.38 ± 0.15	1.53 ± 0.16	1.64 ± 0.6
C16:0	11.46 ± 1.08	8.55 ± 1.67	8.21 ± 1.99	9.00 ± 1.49	11.38 ± 1.65
C16:1, *cis* 9	52.61 ± 4.65	54.84 ± 0.92	55.36 ± 1.50	56.82 ± 1.43	56.80 ± 1.84
C17:0	n.d.	9.86 ± 0.61	5.77 ± 1.39	9.72 ± 1.03	11.48 ± 1.02
C17:1, *cis* 10	n.d.	9.90 ± 2.12	7.82 ± 3.35	n.d.	n.d.
C18:2, *cis* 9,12	n.d.	n.d.	0.05 ± 0.05	0.47 ± 0.29	1.49 ± 0.52
C20:4, *cis* 5,8,11,14	n.d.	0.10 ± 0.10	0.65 ± 0.45	1.65 ± 0.55	2.91 ± 0.72
C20:5, *cis* 5,8,11,14,17	15.59 ± 1.45	5.15 ± 1.22	9.13 ± 1.50	10.72 ± 2.18	10.50 ± 2.77
Total fatty acid	2.10 ± 0.08	1.94 ± 0.05	2.31 ± 0.17	2.42 ± 0.12	2.28 ± 0.11
SFA	30.95 ± 1.66	28.57 ± 1.58	25.52 ± 1.57	28.67 ± 1.67	26.31 ± 1.81
MUFA	53.46 ± 2.33	66.18 ± 2.40	64.65 ± 3.07	58.49 ± 1.36	58.79 ± 1.95
PUFA	15.59 ± 1.45	5.25 ± 1.26	9.84 ± 1.95	12.84 ± 2.21	14.91 ± 2.86

SFA—saturated fatty acids; MUFA—monounsaturated fatty acids; PUFA—polyunsaturated fatty acids; n.d.—not detected.

**Table 7:** The concentration of silicon, nitrogen, and phosphorus and their mass (γ) and molar ratios (C) in modified f/2 growth medium (M1–M6).

Growth Medium	Silicon		Nitrogen		Phosphorus		Ratio Si:N:P^-^	
	γ_Si_ (mg L^−1^)	C_Si_ (mM)	γ_N-NO3_^−^ (mg L^−1^)	C_N-NO3_^−^ (mM)	γ_P-PO4_^3−^ (mg L^−1^)	C_P-PO4_^3−^ (mM)	(g:g:g)	(mol:mol:mol)
M1	14.04	0.5	28.01	2	3.42	0.036	3:8:1	14:56:1
M2	14.04	0.5	28.01	2	13.68	0.15	1:2:1	3:13:1
M3	28.09	1	49.02	3.5	13.68	0.15	2.:4:1	7:23:1
M4	28.09	1	84.04	6	13.68	0.15	2:6:1	7:40:1
M5	28.09	1	49.02	3.5	20.51	0.22	1:2:1	5:16:1
M6	28.09	1	49.02	3.5	27.35	0.29	1:2:1	4:12:1
f/2 *	2.81	0.1	12.32	0.88	3.42	0.036	1:4:1	3:24:1

* original f/2 growth medium.

**Table 8:** Calibration curves of standards analysed using UPLC.

Analyte	Calibration Equation (g L^−1^)	Determination Coefficient
Glucuronic acid	y = 113,192x + 44.361	0.99
Glucose	y = 135,278x − 3377	0.99
Mannose	y = 128,302x − 5039.7	0.99
Galactose	y = 132,077x + 987.09	0.99
Xylose	y = 129,878x − 627.53	0.99
Fructose	y = 125,211x + 3745.8	1.00
Rhamnose	y = 113,900x + 479.24	0.99
Arabinose	y = 128,443x − 3314.1	0.99
Fucose	y = 136,441x − 2965.8	0.99
Glucosamine hydrochloride	y = 128.54x + 279.1	0.98

## Data Availability

The data presented in this study are available for a limited time upon request from the corresponding author.

## Supplementary Materials

The following are available online at www.mdpi.com/xxx/s1, Figure S1: Growth curves of diatom *Nitzschia* sp. at different nitrogen concentrations in f/2 medium: (a) 0 mM; (b) 0.88 mM; (c) 2 mM; (d) 3.5 mM; (e) 6 mM; (f) 8 mM; Figure S2: Growth curves of diatom *Nitzschia* sp. at different silicon concentrations in f/2 medium: (a) 0 mM and 0 mM in plastic flasks; (b) 0.1 mM; (c) 0.5 mM; (d) 1 mM; (e) 1.5 mM; (f) 2 mM; Figure S3: Growth curves of diatom *Nitzschia* sp. at different phosphate concentrations in f/2 medium: (a) 0 mM; (b) 0.036 mM; (c) 0.15 mM; (d) 0.36 mM; Table S1: Consumption of nitrogen, silicon, and phosphate during cultivation of diatom *Nitzschia* sp. S5 with different nutrient concentrations in f/2 medium and their ratios; Table S2: Consumption of nitrogen, silicon, and phosphate during fed-batch cultivation of diatom *Nitzschia* sp. S5.
