# Supplementary material for: Enhancement of Biomass Production of Diatom Nitzschia sp. S5 through Optimisation of Growth Medium Composition and Fed-Batch Cultivation
Source: Mar Drugs. 2024 Jan 17;22(1):0. doi: 10.3390/md22010046 (PMC11154399; doi:10.3390/md22010046)
Supplement: Supplementary file 1 [file marinedrugs-22-00046-s001.zip › marinedrugs-2798331-supplementary.pdf]

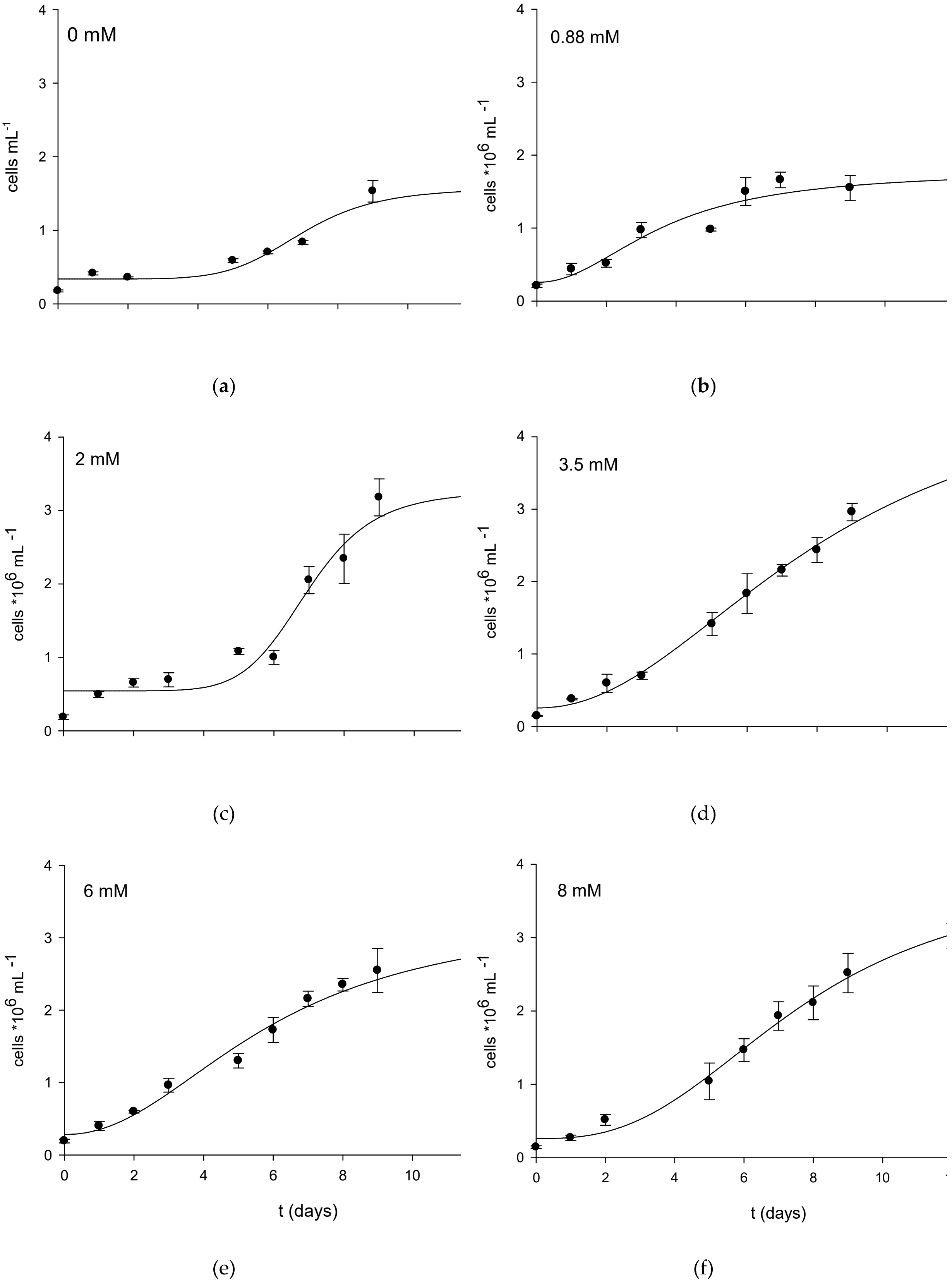

Figure S1. Growth curves of diatom *Nitzschia* sp. S5 grown in f/2 medium with different nitrogen concentrations: (**a**) 0 mM; (**b**) 0.88 mM; **(c)** 2 mM; **(d)** 3.5 mM; **(e)** 6 mM; **(f)** 8 mM.

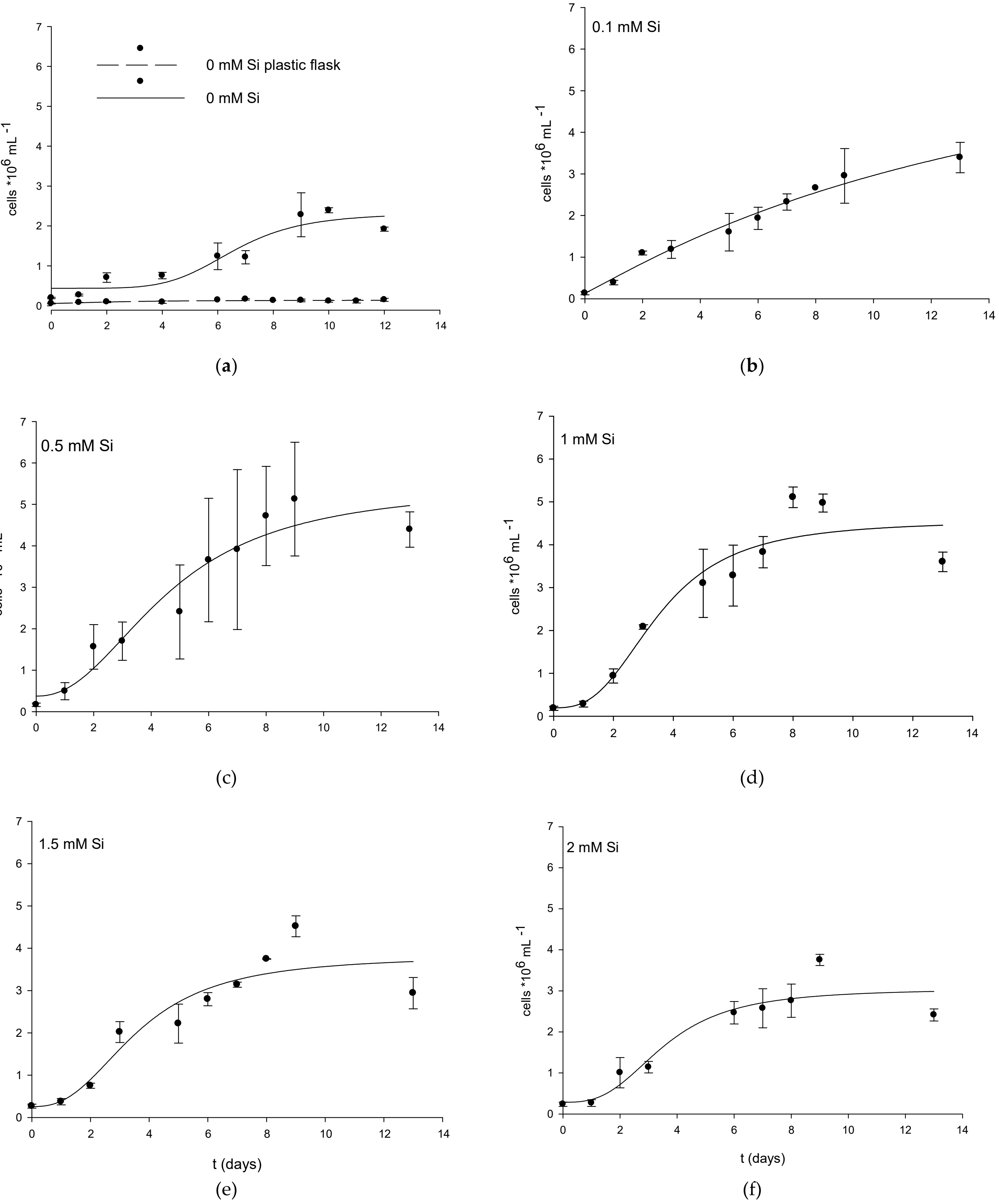

Figure S2. Growth curves of diatom *Nitzschia* sp. S5 in f/2 medium with different silicon concentrations: (**a**) 0 mM in glass and plastic* Erlenmeyer flasks; (**b**) 0.1 mM; **(c)** 0.5 mM; **(d)** 1 mM; **(e)** 1.5 mM; **(f)** 2 mM. *To evaluate the effect of silicon leaching form glass Erlemeyer flask on diatom

growth under sillicon depravation condition the cultivation was conduceted in plastic Erlenmeyer flask.

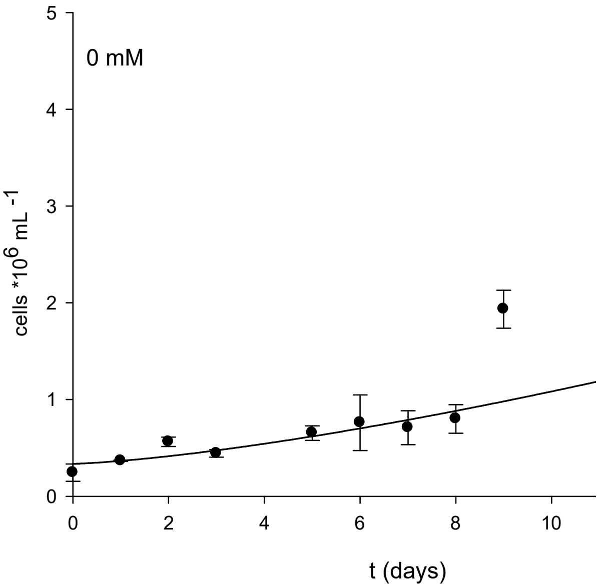

(**a**)

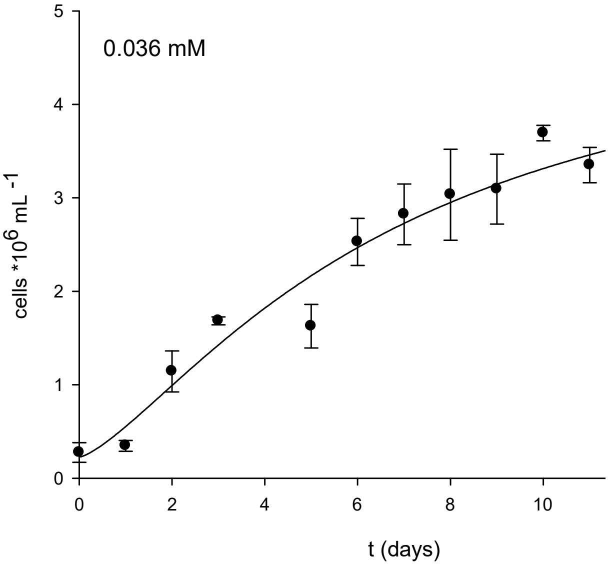

(**b**)

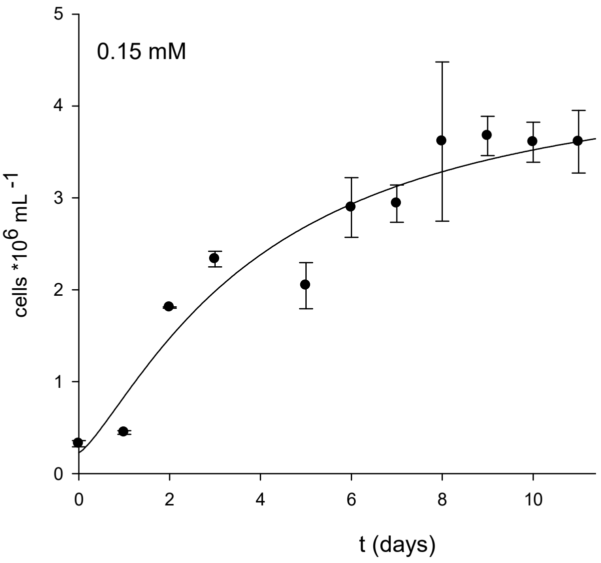

(c)

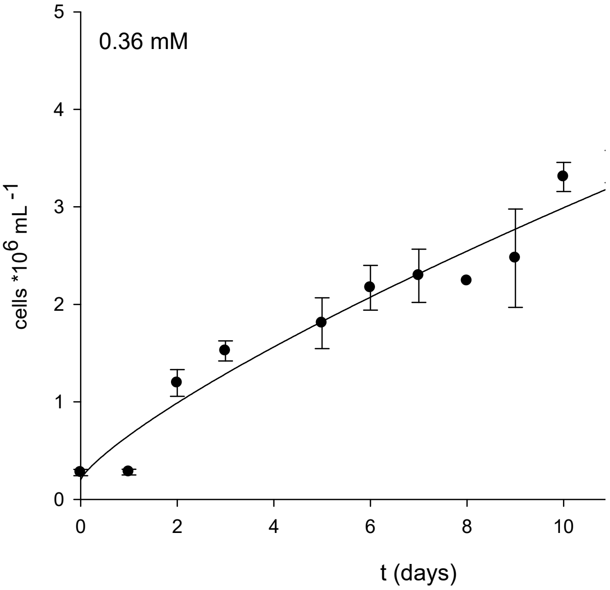

(d)

Figure S3. Growth curves of diatom *Nitzschi*a sp. S5 at different phosphate concentrations in f/2 medium: (**a**) 0 mM; (**b**) 0.036 mM; **(c)** 0.15 mM; **(d)** 0.36 mM.

Table S1. Consumption of nitrogen, silicon and phosphate at beginning and at the end of cultivation of *Nitzschia* sp. S5 with different macronutrient concentrations in f/2 medium and their ratios.

C (mM)		$g_{N\text{-}NO3\text{-}}$ (mg L^{-1})		g_{Si} (mg L^{-1})		$g_{PO4\text{-}}$ (mg L^{-1})	
		t_o	t_{final}	t_o	t_{final}	t_o	t_{final}
$N\text{-}NO^{3-}$	0.00	0	0	2.81*	0.07±0.03	3.42*	0.00±0.00
	0.88	12.32	6.08±0.19		0.19±0.05		0.00±0.00
	2.00	28.01	6.09±0.23		0.01±0.004		0.00±0.00
	3.50	49.02	6.30±0.36		0.30±0.23		0.00±0.00
	6.00	84.04	8.98±1.81		0.99±0.42		0.00±0.00
	8.00	112.05	6.71±0.19		0.97±0.11		0.00±0.00
Si	0.0**	12.32*	29.39±2.28	0	0.05±0.03	3.42*	0.00±0.00
	0.0		1.76±0.20	0	0.04±0.03		0.00±0.00
	0.1		1.99±0.38	2.81	0.17±0.07		0.00±0.00
	0.5		0.91±0.43	14.04	0.37±0.52		0.00±0.00
	1.0		0.67±0.03	28.09	1.56±0.28		0.00±0.00
	1.5		1.04±0.17	42.13	1.32±0.10		0.00±0.00
	2.0		1.69±0.25	56.17	1.22±0.07		0.00±0.00
$PO_4{}^{3-}$	0	12.32*	8.39±0.17	2.81*	0.00±0.00	0.00	0.00±0.00
	0.036		2.25±0.32		0.00±0.00	3.42	0.00±0.00
	0.15		2.99±0.94		0.00±0.00	14.25	0.00±0.00
	036		2.99±1.04		0.04±0.02	34.19	3.79±0.76
Modified f/2							
Ratio 1		28.01	2.67±0.70	11.23	0.20±0.06	3.42	0.04±0.01
Ratio 2		28.01	3.48±0.85	11.23	0.07±0.02	13.68	0.70±0.29
Ratio 3		49.02	3.95±0.92	28.09	0.20±0.13	13.68	0.12±0.05
Ratio 4		84.04	3.48±2.84	28.09	0.20±0.09	13.68	0.07±0.06
Ratio 5		49.02	11.99±1.13	28.09	2.08±1.42	20.51	1.41±0.69
Ratio 6		49.02	8.07±0.56	28.09	2.11±0.47	27.35	1.91±0.15
Control		12.33	8.72±0.42	2.81	0.10±0.08	3.42	0.24±0.12

*same concentration of macronutrient for experimatal set.

**plastic Erlenmeyer flasks were used.

Table S2. Consumption of nitrogen, silicon and phosphate during fed- batch cultivation of diatom *Nitzschia* sp. S5.

Cultivation mode	$\gamma_{N\text{-}NO3\text{-}}$ (mg L^{-1})		γ_{Si} (mg L^{-1})		$\gamma_{PO4\text{-}}$ (mg L^{-1})	
	t_o	t_{final}	t_o	t_{final}	t_o	t_{final}
Batch	49.02	17.23	28.09	0.42	13.68	1.87±0.07
Fed batch						
I nutrient addition	35.83	9.26±0.77	11.62	1.86±0.88	7.48	1.31±0.08
II nutrient addition	27.86	12.50±2.18	13.06	1.12±0.52	6.92	1.33±0.05
III nutrient addition	33.21	14.61±0.79	12.32	3.02±0.78	6.99	1.37±0.05
IV nutrient addition	39.95	21.35±0.38	15.19	3.40±0.23	7.11	1.49±0.18
